# The Relationship between Coronary Flow Reserve and the TyG Index in Patients with Gestational Diabetes Mellitus

**DOI:** 10.3390/medicina59101811

**Published:** 2023-10-12

**Authors:** Serhan Ozyildirim, Hasan Ali Barman, Omer Dogan, Murat Kazim Ersanli, Sait Mesut Dogan

**Affiliations:** Institute of Cardiology, Department of Cardiology, Istanbul University-Cerrahpasa, Istanbul 34320, Turkey; serhanozyildirim@gmail.com (S.O.); omrdgn123@gmail.com (O.D.); mersanli@gmail.com (M.K.E.); smdogan@yahoo.com (S.M.D.)

**Keywords:** gestational diabetes mellitus, coronary flow reserve, triglyceride glucose index

## Abstract

*Background and Objectives*: Gestational diabetes mellitus (GDM) is a prevalent metabolic disorder characterized by glucose intolerance during pregnancy. The triglyceride glucose (TyG) index, a marker of insulin resistance, and coronary flow reserve (CFR), a measure of coronary microvascular function, are emerging as potential indicators of cardiovascular risk. This study aims to investigate the association between CFR and the TyG index in GDM patients. *Materials and Methods*: This cross-sectional study of 87 GDM patients and 36 healthy controls was conducted. The participants underwent clinical assessments, blood tests, and echocardiographic evaluations. The TyG index was calculated as ln(triglycerides × fasting glucose/2). CFR was measured using Doppler echocardiography during rest and hyperemia induced by dipyridamole. *Results*: The study included 87 individuals in the GDM group and 36 individuals in the control group. There was no significant difference in age between the two groups (34.1 ± 5.3 years for GDM vs. 33.1 ± 4.9 years for the control, *p* = 0.364). The TyG index was significantly higher in the GDM group compared to the controls (*p* < 0.001). CFR was lower in the GDM group (*p* < 0.001). A negative correlation between the TyG index and CFR was observed (r = −0.624, *p* < 0.001). Linear regression revealed the TyG index as an independent predictor of reduced CFR. *Conclusions*: The study findings reveal a significant association between the TyG index and CFR in GDM patients, suggesting their potential role in assessing cardiovascular risk.

## 1. Introduction

Gestational diabetes mellitus (GDM) affects approximately 7% of pregnancies worldwide, making it one of the most prevalent medical conditions during gestation. It is characterized by glucose intolerance that develops during pregnancy [1,2]. While GDM is often considered a transient condition that resolves after delivery, emerging evidence suggests that it may have long-lasting implications for both maternal and fetal health [3]. Maternal complications associated with gestational diabetes mellitus (GDM) encompass a heightened risk of developing type 2 diabetes mellitus (T2DM) in the years following pregnancy. Moreover, women with a history of GDM face an elevated risk of cardiovascular diseases (CVDs), including conditions such as hypertension, coronary artery disease, and stroke, which can manifest later in life [4]. CVDs stand as a foremost cause of mortality and morbidity on a global scale. It is of paramount importance to gain a comprehensive understanding of the cardiovascular repercussions associated with GDM, given its potential to offer a unique opportunity to delve into the intricate relationship between metabolic disruptions during pregnancy and subsequent long-term cardiovascular well-being. Recent research has brought to the forefront the potential significance of insulin resistance and metabolic dysfunction as underlying factors contributing to the heightened susceptibility to CVDs in individuals with a history of GDM. This underscores the need for continued investigation and vigilance in managing the cardiovascular health of those who have experienced GDM, as it has implications not only during pregnancy but also in the years that follow [5,6].

The triglyceride glucose (TyG) index, considered a reliable surrogate marker reflecting not only insulin resistance but also beta-cell dysfunction, has gained increasing recognition as a robust predictor for the likelihood of developing future T2DM and CVDs. Its growing importance in clinical research and practice lies in its ability to offer valuable insights into the complex interplay of metabolic factors, making it a valuable tool for assessing the risk of both T2DM and CVDs and ultimately contributing to more proactive and preventive healthcare strategies [7]. The TyG index, calculated as the logarithm of the product of fasting triglyceride and fasting glucose levels, has been shown to correlate with insulin resistance measured by the hyperinsulinemic–euglycemic clamp method [8]. However, the association between the TyG index and cardiovascular function, particularly coronary microvascular function, in patients with GDM remains insufficiently investigated.

Coronary flow reserve (CFR), serving as an index that gauges the functionality of the coronary microvasculature, serves as an indicator of the coronary circulation’s capacity to expand in response to heightened myocardial oxygen demand. [9,10]. In a recent study related to microvascular resistance reserve (MRR) is independent of epicardial resistance, and the lower the fractional flow reserve (FFR) value, the greater the difference between MRR and coronary flow reserve (CFR). Therefore, MRR is proposed as a specific, quantitative, and operator-independent metric for quantifying coronary microvascular dysfunction [11]. CFR has garnered recognition as an early and sensitive marker indicative of subclinical coronary artery disease, serving as an early warning sign of potential cardiovascular issues. Moreover, this impairment in CFR has been consistently linked to adverse cardiovascular outcomes, emphasizing its significance as a valuable prognostic indicator that underscores the importance of monitoring and intervention in individuals at risk of heart-related health complications [12]. Considering the emerging significance of both the TyG index and CFR in assessing cardiovascular risk, investigating their potential relationship in GDM patients may provide valuable insights into the early cardiovascular alterations associated with this metabolic disorder.

This study aims to explore the potential association between CFR and the TyG index in patients with GDM.

## 2. Methods

### 2.1. Study Design

In this cross-sectional study, a comprehensive participant pool was established, encompassing a total of 123 individuals. Among them, 87 participants had received a diagnosis of GDM, while the remaining 36 individuals constituted the healthy control group. The study was meticulously conducted within the controlled environment of a tertiary care university hospital, ensuring the quality and accuracy of the research procedures.

### 2.2. Participant Selection

In this study, eligible participants were limited to women aged over 18 years who had received a confirmed diagnosis of GDM based on the criteria established by the American Diabetes Association [13]. Additionally, the control subjects were selected from pregnant women, matched for age, who exhibited normal glucose tolerance. On the other hand, individuals with a documented history of pre-existing diabetes, cardiovascular diseases, chronic kidney disease, hypertension, or any other substantial medical condition were excluded from the study. These strict inclusion and exclusion criteria were applied to ensure the homogeneity of the study population and the validity of the research findings.

### 2.3. Ethical Considerations

The study adhered to the ethical principles outlined in the Declaration of Helsinki. Ethical approval was obtained from the institutional review board. Informed consent was obtained from all participants before their inclusion in the study.

### 2.4. Clinical and Laboratory Assessments

The clinical assessments in this study involved a meticulous gathering of detailed medical histories from each participant, coupled with comprehensive physical examinations. Within the cohort diagnosed with gestational diabetes mellitus (GDM), specific pregnancy-related characteristics, including gestational age, parity, and obstetric history, were meticulously documented. To obtain fasting blood samples, all participants underwent an overnight fast of at least 8 h. The subsequent biochemical analyses covered an array of critical parameters, including fasting glucose, insulin, triglycerides, total cholesterol, high-density lipoprotein cholesterol (HDL-C), and low-density lipoprotein cholesterol (LDL-C) levels. The TyG index was calculated using the formula: TyG index = ln[fasting triglycerides (mg/dL) × fasting glucose (mg/dL)/2].

### 2.5. Echocardiographic Evaluation and Coronary Flow Reserve Measurement

All echocardiographic examinations were conducted using an ultrasound platform equipped with a matrix-array transducer (Vivid 6, GE Healthcare, Chicago, IL, USA), in accordance with established protocols outlined by the American Society of Echocardiography [14]. These measurements included left ventricular ejection fraction (LVEF), left ventricular end-diastolic diameter (LVEDD), left ventricular end-systolic diameter (LVESD), left ventricular end-diastolic volume (LVEDV), left ventricular end-systolic volume (LVESV), stroke volume, left ventricular septal wall thickness, left ventricular posterior wall thickness, left ventricular mass index, left atrium size, E-wave velocity (E), A-wave velocity (A), E/A ratio, mitral deceleration time (MDT), ejection time (ET), isovolumetric relaxation time (IVRT), and various tissue Doppler measurements.

Doppler echocardiography was employed to evaluate CFR. Then participants were instructed to abstain from caffeine-containing products for at least 24 h before the examination. To assess coronary flow measurements, we visualized the distal portion of the left anterior descending artery by utilizing a high-frequency ultrasound beam. Color Doppler gain optimization was achieved through conventional techniques, and the Nyquist limit was set within the range of 0.16–0.50 m/s. After vessel visualization, a pulse-wave Doppler cursor was strategically positioned to measure coronary flow velocity. The measurements were obtained both before and after the administration of dipyridamole infusion (0.84 mg/kg for 6 min), a standard pharmacological stressor inducing hyperemia. Peak diastolic flow velocity was measured in the left anterior descending coronary artery during both at rest and hyperemia. CFR was calculated as the ratio of hyperemic peak diastolic flow velocity to baseline peak diastolic flow velocity [15,16].

### 2.6. Statistical Analyses

All statistical tests were conducted using the Statistical Package for the Social Sciences 25.0 for Windows (SPSS Inc., Chicago, IL, USA). The Kolmogorov–Smirnov test was used to analyze the normality of the data. Normally distributed numerical data were expressed as the mean ± SD, non-normally distributed parameters were expressed as median (25–75) percentiles, while categorical data were expressed as percentages. The relationships among the parameters were assessed using Pearson’s or Spearman’s correlation analysis according to the normality of the data. Student’s *t*-test or the Mann–Whitney U test was used to compare unpaired samples as needed. Correlations between variables were evaluated by Pearson’s rank correlation test. Multiple linear regression analyses using the stepwise method were performed to assess the independent variables affecting the dependent variable CFR. All independent variables in the multiple linear regression were tested for multicollinearity. If the variance inflation factor (VIF) exceeded 3.0, the variable was considered to be collinear. All reported confidence interval (CI) values were calculated at the 95% level. Significance was assumed at a 2-sided *p* < 0.05.

The sample size was determined using the G-power program based on effect size (Supplementary Materials), type 1 error, and study power. The type 1 error rate was set at 5%, and the study power was set at 80%, while the effect size was determined from previous studies in the literature.

## 3. Results

**Figure 1:**
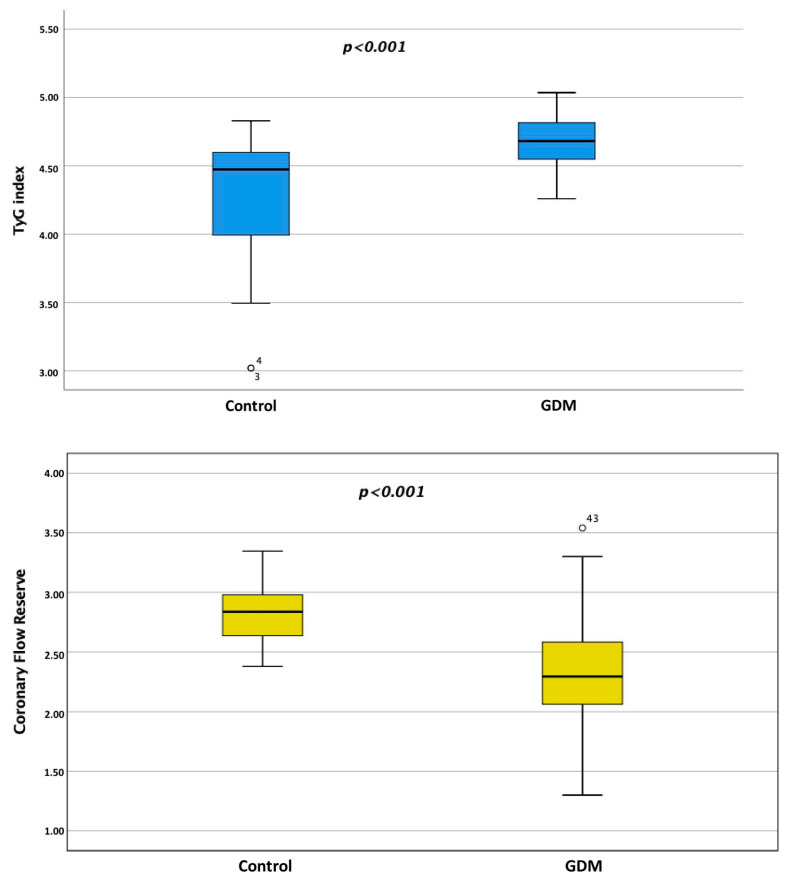
The TyG index was significantly higher, and CFR was significantly lower in the GDM group compared to the control group.

The demographic and clinical characteristics of patients diagnosed with GDM and the control group are summarized in Table 1. The study included 87 individuals in the GDM group and 36 individuals in the control group. No statistically significant difference was observed in age between the two groups (34.1 ± 5.3 years for GDM vs. 33.1 ± 4.9 years for the control, *p* = 0.364). The gestational age was 33.3 ± 4.4 years for the GDM group. The gender distribution was consistent among both groups, with 100% female participants. Body mass index (BMI) values were comparable between the GDM and control groups (26.7 ± 3.4 vs. 26.1 ± 2.7, *p* = 0.407). Body surface area (BSA) was slightly lower in the GDM group (1.81 ± 0.16) compared to the control group (1.88 ± 0.15, *p* = 0.036). Systolic blood pressure (SBP) and diastolic blood pressure (DBP) showed no statistically significant difference between the two groups. Heart rate was similar in both groups (72.4 ± 4.3 for GDM vs. 72.6 ± 4.5 for the control, *p* = 0.776). The TyG index was significantly higher in the GDM group compared to the control group (4.6 ± 0.1 vs. 4.3 ± 0.4, *p* < 0.001). Fasting blood glucose (FBG) and postprandial blood glucose (PBG) levels did not significantly differ between the two groups. HbA1C levels were higher in the GDM group (5.3 ± 0.3) compared to the control group (4.9 ± 0.1, *p* < 0.001). Homeostatic model assessment for insulin resistance (HOMA-IR) and uric acid levels showed no significant differences between the groups. C-reactive protein (CRP) levels were slightly elevated in the GDM group (2.6, interquartile range 1.3–4.2) compared to the control group (1.2, interquartile range 0.9–3.5, *p* = 0.066). Hemoglobin (Hgb), total cholesterol (TC), low-density lipoprotein (LDL) cholesterol, high-density lipoprotein (HDL) cholesterol, and triglyceride levels did not display statistically significant differences between the groups (Table 1, Figure 1).

Table 2 illustrates the comparison of conventional echocardiographic parameters between the two groups. The study included 87 participants in the GDM group and 36 participants in the control group. The parameters assessed include left ventricular ejection fraction (LVEF), left ventricular end-diastolic diameter (LVEDD), left ventricular end-systolic diameter (LVESD), left ventricular end-diastolic volume (LVEDV), left ventricular end-systolic volume (LVESV), stroke volume, left ventricular septal wall thickness, left ventricular posterior wall thickness, left ventricular mass index, left atrium size, E and A wave velocities, E/A ratio, mitral deceleration time (MDT), ejection time (ET), isovolumic relaxation time (IVRT), lateral E’ and A’ velocities, lateral E’/A’ ratio, lateral S wave velocity, lateral IVCT (isovolumic contraction time), lateral IVRT (isovolumic relaxation time), lateral ET (ejection time), basal diastolic blood flow velocity (DBFV), hyperemic diastolic blood flow velocity, and CFR. No statistically significant differences were observed for most parameters between the groups, except for the A wave velocity, E/A ratio, MDT, ET, lateral A’ velocity, lateral IVCT, lateral E’/A’ ratio, basal DBFV, hyperemic DBFV, and CFR, where the differences were found to be significant (*p* < 0.05). These findings provide insights into the echocardiographic differences in these two groups (Table 2).

The CFR parameters were assessed using linear regression models. CFR-related parameters and statistically significant variables were included in the regression analysis. The associations of CFR with hemoglobin, LV mass index, uric acid, HBA1C, BMI, CRP, TC, SBP, age, and the TyG index were assessed in the regression analysis. The TyG index, HbA1C, and TC were independent predictors of a reduced CRF values (Table 3).

**Figure 2:**
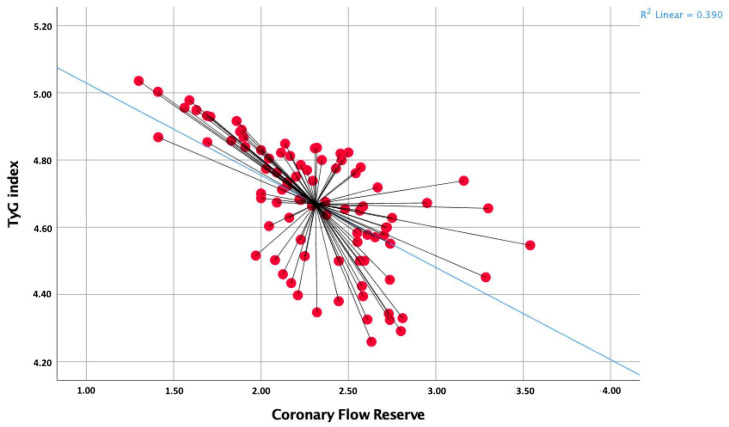
Correlation analysis between the CFR and the TyG index.

The Pearson correlation analysis revealed a significant negative correlation between CFR values and the TyG index (r = −0.624, *p* < 0.001, Figure 2).

## 4. Discussion

In this study, we explored the potential association between CFR and the TyG index among patients with GDM. The key findings of our investigation are as follows: (i) the TyG index was significantly higher in the GDM group compared to the control group (4.6 ± 0.1 vs. 4.3 ± 0.4, *p* < 0.001); (ii) CFR was significantly lower in the GDM group compared to the control group (2.3 ± 0.4 vs. 2.8 ± 0.2, *p* < 0.001); (iii) our regression analysis revealed that the TyG index independently predicted diminished CFR values; and (iv) we observed a highly significant negative correlation between the TyG index and CFR values (r = −0.624, *p* < 0.001).

GDM is characterized as a transient and pregnancy-induced form of diabetes that emerges during the gestational period. It is noteworthy for being accompanied by a spectrum of diverse metabolic alterations and fluctuations in physiological processes, making it a distinct and specific type of diabetes that primarily manifests during pregnancy [17]. While historically viewed as a temporary condition confined to the duration of pregnancy, emerging evidence is increasingly pointing towards potential implications of gestational diabetes mellitus (GDM) on long-term cardiovascular health [18]. In recent years, the TyG index has risen to prominence as a valuable and reliable indicator of not only insulin resistance but also metabolic dysfunction, as evidenced by a growing body of research [19,20]. Likewise, coronary flow reserve (CFR), serving as an index that reflects the functionality of the coronary microvasculature, has gained increased recognition as an early and sensitive marker of cardiovascular dysfunction, further emphasizing its importance in the realm of cardiovascular health assessment [21].

Past studies have shown reduced coronary flow velocity reserve (CFVR) and increased epicardial fat thickness (EFT) in women with a history of GDM, suggesting an elevated risk of coronary microvascular dysfunction [22]. A recent study highlighted that women with both a history of preeclampsia (pPE) and GDM exhibited reduced CFVR and an augmented risk of CMD, underlining the combined impact of these conditions on cardiovascular risk [23]. Moreover, previous research has demonstrated a link between adverse pregnancy outcomes and lower CFR in women with ischemic symptoms [24]. Examining the association between the TyG index and GDM, a meta-analysis reported a four-fold increase in TyG index levels in GDM patients [25]. In a study aiming to assess the relationship between the TyG index and pregnancy-related complications, the TyG index during the first trimester was significantly associated with the risk of gestational diabetes [26]. In a recent study, coronary microvascular function was compared between diabetic and non-diabetic patients. The study utilized continuous intracoronary thermodilution to assess various parameters, including coronary flow reserve (CFR) and microvascular resistance reserve (MRR). Additionally, it examined left atrial reservoir strain (LASr), an early indicator of diastolic dysfunction. This study emphasizes that the indexes obtained through continuous intracoronary thermodilution enable a reliable and operator-independent evaluation of both coronary macro- and micro-vessels [27]. In contrast to earlier studies that explored CFR and the TyG index separately in relation to GDM, our study uniquely examined both parameters together. Our findings, consistent with previous research, indicated an elevated TyG index and reduced CFR levels in GDM patients. However, our study further unveiled the independent predictive value of the TyG index for CFR levels.

Our primary finding highlights the clinical relevance of the TyG index as a marker of metabolic perturbation in GDM. The elevated TyG index in the GDM group aligns with its established role in indicating insulin resistance and beta-cell dysfunction [28]. This supports the idea that GDM involves systemic metabolic disruptions extending beyond pregnancy, contributing to long-term cardiovascular risk. Furthermore, the reduced CFR among GDM patients signifies a possible connection between gestational hyperglycemia and impaired coronary microvascular function [29]. The diminished CFR values indicate compromised coronary vasodilatory capacity, often linked with early coronary artery disease and adverse cardiovascular outcomes [30]. This corresponds with a growing body of research indicating heightened cardiovascular risk in GDM patients. Our study, uniquely, demonstrates the TyG index’s independent predictive value for diminished CFR. This highlights the clinical relevance of the TyG index as an accessible tool for identifying GDM patients at heightened cardiovascular risk. By serving as an early marker of compromised coronary microvascular function, the TyG index could aid in risk stratification and guide targeted interventions to mitigate long-term cardiovascular complications in this population [31].

Critically, the substantial and robust negative correlation that has been discerned between the TyG index and CFR values underlines and amplifies the potential significance of the TyG index as an encompassing marker for the comprehensive evaluation of cardiovascular risk within the context of GDM. This inverse relationship not only accentuates but also underscores the intricate and multifaceted interplay between metabolic health parameters and the functionality of the coronary microvasculature, particularly in the unique setting of GDM.

The mechanistic underpinnings of this observed correlation demand further scrutiny and exploration. Delving into the molecular pathways that connect metabolic dysfunction, as indicated by the TyG index, with the impairment seen in coronary microvascular function, as measured by CFR, holds considerable promise for advancing our understanding of the intricate pathophysiological mechanisms at play. Such insights may not only refine our comprehension of GDM-related cardiovascular risk but also potentially inform novel therapeutic strategies and preventive interventions for individuals grappling with this condition.

While our study offers valuable insights into the association between the TyG index and CFR within the context of GDM patients, it is imperative to recognize and acknowledge certain inherent limitations. First and foremost, the cross-sectional design of our study precludes us from establishing causality, and therefore, it is crucial to emphasize the necessity for future longitudinal investigations that can provide a more dynamic perspective on these relationships. Furthermore, it is important to exercise caution in generalizing our findings due to the relatively modest sample size and the single-center focus of our study. These limitations underscore the importance of further research endeavors that encompass larger, more diverse cohorts and multi-center studies, which may yield a more comprehensive understanding of the intricate interplay between the TyG index and CFR in the context of GDM.

## 5. Conclusions

In conclusion, our comprehensive investigation has unveiled the TyG index as a potentially pivotal and pragmatic marker for assessing cardiovascular risk in the specific cohort of individuals diagnosed with GDM. The intricate connection observed between the TyG index and CFR beckons us to reevaluate the conventional paradigms of risk assessment and recognition of the multifaceted interplay between metabolic parameters and cardiovascular health within the context of GDM.

However, it is vital to recognize that this study serves as a foundational cornerstone, prompting further inquiries into uncharted territories. Future investigations should embark on a more profound exploration of the intricate mechanisms underpinning the association between the TyG index and CFR. This inquiry should extend to examining whether the TyG index can be leveraged as a clinically meaningful tool for tailoring interventions aimed at mitigating and managing the long-term cardiovascular complications that may afflict GDM patients. In essence, our findings beckon us to envision a future where personalized healthcare strategies, informed by the TyG index, offer enhanced and tailored cardiovascular risk management for individuals navigating the complexities of GDM. This journey toward a more holistic understanding and proactive approach to cardiovascular health is poised to improve the well-being and outcomes of this patient population, representing a noteworthy stride in the realm of diabetes and cardiovascular research.

## Figures and Tables

**Table 1:** Demographic and clinical characteristics of patients diagnosed with gestational diabetes mellitus and the control group.

	GDM (*n* = 87)	Control (*n* = 36)	*p*
Age (years)	34.1 ± 5.3	33.1 ± 4.9	0.364
Gestational age (years)	33.3 ± 4.4	-	-
Gender, female n (%)	87 (%100)	36 (%100)	-
BMI, kg/m^2^	26.7 ± 3.4	26.1 ± 2.7	0.407
BSA (m^2^)	1.81 ± 0.16	1.88 ± 0.15	0.036
SBP (mmHg)	119.9 ± 8.9	116.6 ± 11.8	0.098
DBP (mmHg)	75.8 ± 4.9	73.8 ± 7.6	0.155
Heart rate (beats/min)	72.4 ± 4.3	72.6 ± 4.5	0.776
TyG index	4.6 ± 0.1	4.3 ± 0.4	<0.001
FBG (mg/dL)	93.5 ± 7.0	92.9 ± 4.5	0.681
PBG (mg/dL)	110.5 ± 15.1	115.5 ± 11.8	0.093
HBA1C (mmol/L %)	5.3 ± 0.3	4.9 ± 0.1	<0.001
HOMA-IR	2.7 ± 1.4	2.4 ± 1.0	0.216
Uric acid (μmol/L)	4.8 ± 1.2	4.2 ± 1.4	0.023
CRP (mg/L)	2.6 (1.3–4.2)	1.2 (0.9–3.5)	0.066
Hgb (g/dl)	13.8 ± 2.8	14.1 ± 1.3	0.676
TC (mg/dL)	193.0 ± 24.3	178.1 ± 29.8	0.005
LDL (mg/dL)	121.6 ± 21.1	108.3 ± 27.4	0.005
HDL (mg/dL)	45.5 ± 8.9	45.6 ± 10.2	0.999
Triglyceride (mg/dL)	131.0 ± 45.6	122.5 ± 57.4	0.391

Abbreviations: GDM, gestational diabetes mellitus; BMI, body mass index; BSA, body surface area; SBP, systolic blood pressure; DBP, diastolic blood pressure; TyG index, triglyceride glucose index; FBG, fasting blood glucose; PBG, postprandial blood glucose; HBA1C, hemoglobin A1C; HOMA-IR: homeostasis model assessment of insulin resistance; CRP, C-reactive protein; Hgb, hemoglobin; TC, total cholesterol; LDL, low-density lipoprotein; HDL, high-density lipoprotein.

**Table 2:** Comparison of conventional echocardiographic parameters of the groups.

	GDM (*n* = 87)	Control (*n* = 36)	*p*
LVEF (%)	62.1 ± 5.0	61.7 ± 3.4	0.705
LVEDD (cm)	45.3± 4.1	44.8 ± 3.3	0.522
LVESD (cm)	28.3 ± 2.9	28.0± 2.2	0.700
LVEDV (mL)	137.8 ± 25.8	134.3 ± 20.8	0.468
LVESV (mL)	51.9 ± 11.3	51.2 ± 8.8	0.719
Stroke volume	85.8 ± 18.9	83.0 ± 14.6	0.432
LV—septal wall (cm)	8.9± 0.9	8.9 ± 1.0	0.931
LV—posterior wall (cm)	8.4± 1.2	8.5 ± 0.8	0.642
LV mass index	71.3 ± 15.2	67.6 ± 13.7	0.212
Left atrium (mm)	31.9 ± 3.4	30.8 ± 3.4	0.101
E (cm/s)	81.8 ± 11.9	81.5 ± 6.8	0.858
A (cm/s)	70.2 ± 13.2	62.0 ± 7.9	0.001
E/A ratio	1.1 ± 0.2	1.3 ± 0.1	0.003
MDT	196.4 ± 28.9	184.5 ± 22.3	0.031
ET	5.4 ± 1.3	4.3 ± 1.1	<0.001
IVRT	104.2 ± 11.7	96.4 ± 14.0	0.002
Lateral E’ (cm/s)	20.7 ± 4.0	19.7 ± 4.6	0.239
Lateral A’ (cm/s)	17.1 ± 2.9	14.6 ± 2.0	<0.001
Lateral E’/A’ ratio	1.2 ± 0.2	1.3 ± 0.1	0.022
Lateral S (cm/s)	15.7 ± 3.3	14.6 ± 3.0	0.108
Lateral IVCT (ms)	53.7 ± 10.4	47.7 ± 6.0	0.002
Lateral IVRT (ms)	95.5 ± 16.8	95.0 ± 12.6	0.854
Lateral ET	269.2 ± 21.4	274.7 ± 21.8	0.208
Basal DBFV	26.1 ± 5.9	23.0 ± 5.0	0.007
Hyperemic DBFV	59.2 ± 9.3	65.1 ± 15.3	0.011
CFR	2.3 ± 0.4	2.8 ± 0.2	<0.001

Abbreviations: LVEF, left ventricular ejection fraction; LVEDD, left ventricular end-diastolic diameter; LVESD, left ventricular end-systolic diameter; LVEDV, left ventricular end-diastolic volume; LVESV, left ventricular end-systolic volume; MDT, mitral deceleration time; ET, ejection time; IVRT, isovolumic relaxation time; IVCT, isovolumic contraction time; DBFV, diastolic blood flow velocity; CFR, coronary flow reserve.

**Table 3:** Independent factors affecting CFR in GDM patients in the stepwise multiple linear regression analysis.

Coefficients ^a^				
**Model**	**Unstandardized** **Coefficients**		**Standardized** **Coefficients**	***p*** **-value**
	**B**	**Std. Error**	**Beta**	
(Constant)	7.200	0.619		<0.001
TyG index	−0.649	0.114	−0.496	<0.001
HbA1c	−0.232	0.100	0.200	0.023
TC	−0.003	0.001	−0.179	0.035
Excluded Variables ^b^				
**Model**	**B**	**Partial** **Correlation**	**Collinearity** **Statistics**	***p-*** **value**
			**Tolerance**	
Age	−0.088	−0.115	0.970	0.288
Hemoglobin	0.081	0.105	0.965	0.331
LV mass index	−0.021	−0.028	0.945	0.800
Uric acid	0.060	0.075	0.880	0.491
BMI	0.075	0.094	0.900	0.387
CRP	0.099	0.122	0.860	0.262
SBP	−0.115	−0.148	0.935	0.172
Lateral E/A	0.016	0.021	0.909	0.850
MDT	−0.019	−0.024	0.897	0.823
IVRT	0.014	0.017	0.763	0.879
Lateral IVCT	−0.074	−0.096	0.949	0.376

^a^. Dependent variable: CFR. ^b^. Correlates in the model: (constant), TyG index. Abbreviations: TyG index, triglyceride-glucose index; HbA1c, hemoglobin A1c; TC, total cholesterol; BMI, body mass index; CRP, c-reactive protein; SBP: systolic blood pressure; MDT, mitral deceleration time; IVRT, isovolumic relaxation time; IVCT, isovolumic contraction time.

## Data Availability

Not applicable.

## Supplementary Materials

The following supporting information can be downloaded at: https://www.mdpi.com/article/10.3390/medicina59101811/s1.
