# Supplementary figures and images for: The Relationship between Coronary Flow Reserve and the TyG Index in Patients with Gestational Diabetes Mellitus
Source: Medicina (Kaunas). 2023 Oct 12;59(10):1811. doi: 10.3390/medicina59101811 (PMC10608421; doi:10.3390/medicina59101811)

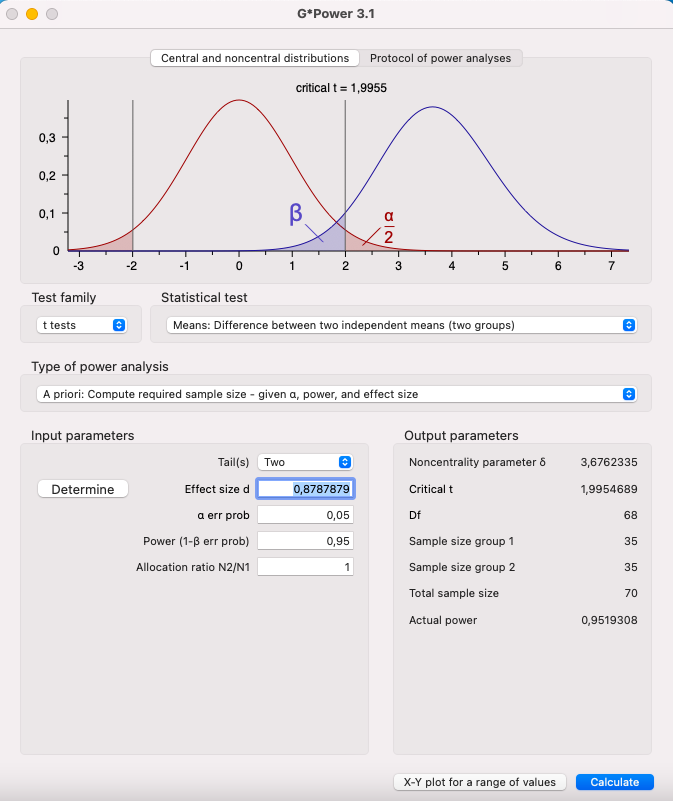

Supplement: Supplementary file 1 [file medicina-59-01811-s001.zip › medicina-2596064-supplementary.png]
